# How Sweet Are Our Gut Beneficial Bacteria? A Focus on Protein Glycosylation in *Lactobacillus*

**DOI:** 10.3390/ijms19010136

**Published:** 2018-01-03

**Authors:** Dimitrios Latousakis, Nathalie Juge

**Affiliations:** Quadram Institute Bioscience, The Gut Health and Food Safety Institute Strategic Programme, Norwich Research Park, Norwich NR4 7UA, UK; Dimitris.Latousakis@quadram.ac.uk

**Keywords:** protein glycosylation, gut commensal bacteria, *Lactobacillus*, glycoproteins, adhesins, lectins, *O*-glycosylation, *N*-glycosylation, probiotics

## Abstract

Protein glycosylation is emerging as an important feature in bacteria. Protein glycosylation systems have been reported and studied in many pathogenic bacteria, revealing an important diversity of glycan structures and pathways within and between bacterial species. These systems play key roles in virulence and pathogenicity. More recently, a large number of bacterial proteins have been found to be glycosylated in gut commensal bacteria. We present an overview of bacterial protein glycosylation systems (*O*- and *N*-glycosylation) in bacteria, with a focus on glycoproteins from gut commensal bacteria, particularly Lactobacilli. These emerging studies underscore the importance of bacterial protein glycosylation in the interaction of the gut microbiota with the host.

## 1. Introduction

Protein glycosylation, i.e., the covalent attachment of a carbohydrate moiety onto a protein, is a highly ubiquitous protein modification in nature, and considered to be one of the post-translational modifications (PTM) targeting the most diverse group of proteins [1]. Although it was originally believed to be restricted to eukaryotic systems and later to archaea, it has become apparent nowadays that protein glycosylation is a common feature in all three domains of life. In fact, it is now believed that at least 70% of eukaryotic and 50% of prokaryotic proteins are glycosylated by post-translational modification [2]. Similar to eukaryotic glycosylation, bacterial glycoproteins can be modified primarily on Asp (*N*-glycosylation) or Ser/Thr (*O*-glycosylation). However, in contrast to eukaryotic glycosylation, where *N*-glycans are pre-assembled onto a lipid carrier before being transferred onto the acceptor protein and *O*-glycans are synthesized directly onto the acceptor protein, bacterial glycosylation is more diverse, both in terms of mechanisms and carbohydrate structures. In addition, while glycosylation in Eukaryotes occurs co-, as well as post-translationally, glycosylation in Prokaryotes is believed to occur post-translationally.

## 2. Overview of Protein Glycosylation in Prokaryotes

### 2.1. N-Glycosylation: The Campylobacter jejuni Paradigm

**Figure 1:**
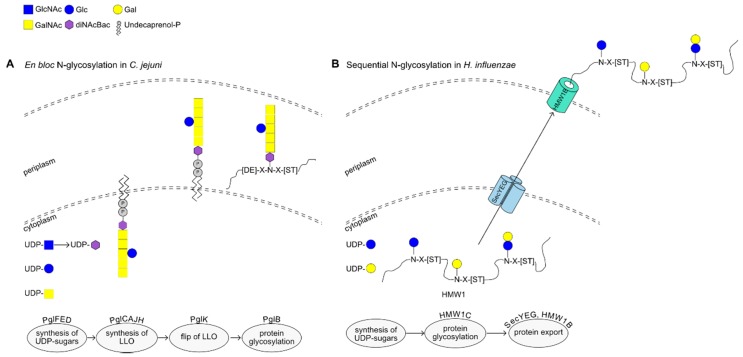
Examples of *N*-glycosylation mechanisms in bacteria. (**A**) *En blocN*-glycosylation in *Campylobacter jejuni* 81–176. (**B**) *N*-glycosylation by sequential addition of monosaccharides in *Haemophilus influenzae* strain 12.

The first complete glycosylation system identified in bacteria, and indeed the best characterized so far, is the one discovered in *Campylobacter jejuni*. *C. jejuni* harbors a protein glycosylation cluster (*pgl*) of 13 genes [3,4] that are responsible for the glycosylation of various proteins [5]. These genes encode (i) enzymes that synthesize bacilosamine (diNAcBac; 2,4-diacetamido-2,4,6-trideoxyglucose) found at the reducing end of the glycan, (ii) glycosyltransferases (GTs) that are involved in the production of the glycan {α-GalNAc-(1,4)- α-GalNAc-(1,4)- [β-Glc-(1,3)-]- α-GalNAc-(1,4)- α-GalNAc-(1,4)- α-GalNAc-(1,3)-α-diNAcBac} on undecaprenol-phosphate, (iii) a transporter (PglK) that flips the glycan to the periplasm and (iv) an oligosaccharyl-transferase (PglB) that glycosylates the target protein [3] (see Figure 1A).

Homologous *pgl* clusters are also found in δ- and ε-proteobacteria, although they vary in terms of their organization and number of GTs. For example, some *Campylobacter* and *Helicobacter* species contain two putative copies of *pglB* whereas the *pgl* cluster in *Helicobacter canadensis* MIT 98-5491 is spread across multiple loci [6].

The *pgl* system resembles the eukaryotic *N*-glycosylation pathway with regards to the glycan synthesis onto a lipid carrier, the requirement for a flippase and the *en bloc* transfer of the glycan onto the target protein. In addition, PglB glycosylates the acceptor protein at an Asp/Glu-Tyr-Asp-Xxx-Ser/Thr motif, similar to that found in eukaryotic glycoproteins. PglB has been shown to have a relaxed specificity towards the oligosaccharide it can transfer [7,8]. The nature of the monosaccharides does not seem to restrict transfer, as heterologous expression of this *N*-glycosylation system in *Escherichia coli* has been used to successfully modify proteins with eukaryotic-like glycans [9]. PglB can also accommodate glycans of varying size, as shown in *E. coli* using *O*-antigen-derived glycans of various lengths [7].

Since this type of glycosylation takes place in the periplasm, and requires flipping of the lipid-linked glycan across the inner membrane, this *N*-glycosylation system has not been identified or predicted in Gram-positive species.

### 2.2. Alternative N-Glycosylation in β- and γ-Proteobacteria

In contrast to the “typical” *N*-glycosylation system found in *Campylobacter* and other δ- and ε-proteobacteria, a different *N*-glycosylation system has been reported in β- and γ-proteobacteria. In particular, the High Molecular Weight Protein 1 (HMW1), an adhesin in *Haemophilus influenza* (*Hi*), was found to undergo *N*-glycosylation in the cytoplasm by HMW1C with one or two hexose (Hex) molecules at over 30 glycosylation sites [10]. Uniquely, HMW1C can perform two distinct reactions, i.e., create an *N*-glycosidic bond between the first Hex and the acceptor protein, and extend the glycan by generating an *O*-glycosidic bond with a second Hex [11] (see Figure 1B). Although HMW1C-like proteins are predicted to exist in many families of β- and γ-proteobacteria [12], it is still unknown if they glycosylate more than one protein. *Hi*HMW1C is involved in the glycosylation of HMW1 while HMW1C-like GTs from *Kingella kingae* and *Aggregatibacter aphrophilus* were shown to glycosylate the trimeric autotransporters Knh and EmaA, respectively. HMW1, Knh and EmaA contain an extended signal peptide of the Type V secretion system, as well as autotransporter-like domains. The HMW1C homologue in *Actinobacillus pleuropneumoniae* is an exception, as it was shown to glycosylate two trimeric autotransporter proteins instead of one [13], with one acceptor protein lacking the Type V secretion system extended peptide. Regarding sugar specificity, *Hi*HMW1C was shown to initiate glycosylation with either glucose (Glc) or galactose (Gal), but further extension with Glc or Gal was only observed when Glc was the monosaccharide at the reducing end [10]. In addition, *Ap*HMW1C was able to utilize UDP-Glc and UDP-Gal as sugar donors, as well as UDP-xylose and GDP-Glc, in an in vitro assay [14]. Although HMW1 glycosylates proteins preferably at the consensus sequon Asn-Xxx-Thr/Ser, different amino acids can be tolerated in the third position [11,14,15]. Recently, the *A. pleuropneumoniae* glycosylation operon was used to add *N*-linked glycan consisting of 1–29 hexose units onto an acceptor protein in *E. coli*, illustrating the potential biotechnological application of this glycosylation system for glycoengineering [16].

### 2.3. N-Glycosylation in Mycoplasmas

While the family of enzymes involved in cytoplasmic *N*-glycosylation appears to be restricted to limited classes of Gram-negative proteobacteria, similar glycosylation mechanisms cannot be excluded from Gram-positive bacteria. In fact, evidence for *N*-glycosylation in mycoplasma species has emerged. In a recent study, Asn and Gln residues outside of the *N*-glycosylation consensus sequence were found to carry a single Hex in *Mycoplasma pulmonis* and *Mycoplasma arthritis* glycoproteins, which could suggest a similar glycosylation mechanism to the one found in *Haemophilus infuenzae* [17]. However, no intracellular glycoproteins could be identified, suggesting that this process may take place extracellularly. In addition, it was shown that the bacteria could use free oligosaccharides from the growth media, without the need to synthesize glycans internally to use for protein glycosylation [17], in support of this mechanism.

### 2.4. O-Glycosylation in Bacteria

**Figure 2:**
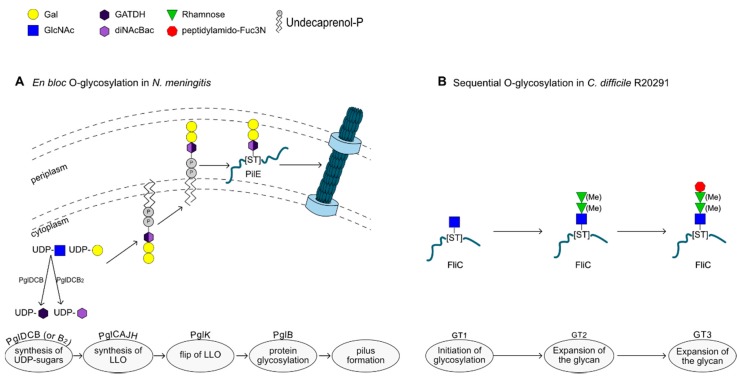
Examples of *O*-glycosylation mechanisms in bacteria. (**A**) *En bloc O*-glycosylation in *Neisseria meningitis*. (**B**) Sequential *O*-glycosylation in *Clostridium difficile* R20291.

Similar to Eukaryotes, bacteria also have mechanisms to perform *O*-glycosylation by modifying protein targets with glycans on Ser or Thr residues, and, as with prokaryotic *N*-glycosylation, two mechanisms have been identified: (i) *en bloc* transfer of a pre-assembled lipid-linked oligosaccharide, and (ii) modification of the acceptor protein directly, by the sequential action of GTs [18] (see Figure 2). The *en bloc* glycosylation mechanism follows a sequence similar to that of the *N*-glycosylation system, i.e., the glycan is synthesized on undecaprenol phosphate, flipped over to the periplasmic space and transferred onto the acceptor protein by the action of an *O*-oligosaccharyltransferase (*O*-OTase). In sequential *O*-glycosylation, multiple GTs act directly onto the acceptor protein to extend the glycan, using sugar nucleotides as donors.

### 2.5. En Bloc O-Glycosylation

Several Gram-negative species have been identified to harbor genes encoding *O*-OTase, including *Neisseria*, *Pseudomonas*, *Aeromonas* and *Burkholderia* spp. [19]. The best studied example of *en bloc O*-glycosylation is that of *Neisseria gonorhoeae*, where PglO, the active *O*-OTase, glycosylates multiple proteins with an *O*-acetylated (OAc) glycan, OAc-Gal-Gal-diNAcBac (see Figure 2A) [20,21,22].

Often, *O*-OTases utilize lipid-linked glycans used in *O*-antigen biosynthesis [23], as with PilO from *Pseudomonas aeruginosa* [24,25] or *Francisella tularensis* [26] for example. To do so, the *O*-antigen subunit is built onto an undecaprenyl phosphate lipid carrier on the cytosolic side of the inner membrane, as in the case of *N*-glycosylation, by the sequential action of a varying number of GTs. It is then flipped across the membrane to the periplasmic space by the transmembrane flippase Wzx. At this stage, the *O*-antigen polymerase Wzy forms a glycosidic bond to link two subunits together [27], or the *O*-OTase uses the synthesized subunit to transfer the glycan onto a glycoprotein [23]. *N. gonorrhoeae* glycoproteins were found to be modified in low-complexity regions (LCRs), rich in Ala, Ser and Pro residues [22], suggesting that some structural features may be recognized by *O*-OTases, although no consensus sequence has been identified. Interestingly, two *O*-OTases were identified in *Acinetobacter baylyi* ADP1, one being specific to pilin glycosylation, whereas the other one could target several proteins [28].

Protein *O*-glycosylation has also been confirmed in *Neisseria elongata* subsp. *glycolytica* [29], a facultative pathogenic, oral bacterial species. However, while *N. gnorrhoeae* and *Neisseria meningitides* bacteria [30] produce a diNacBac-Gal-Gal trisaccharide, by the subsequent action of PglA and PglE, *N. elongata* lacks the genes encoding these enzymes, and instead uses other GTs to generate diNacBac-Glc-di-*N*-acetyl-hexuronic acid (diNAcHexA)-HexNAc. The glycosylation process occurs in the periplasm in an *en bloc* glycosylation manner.

Most of these systems have been studied in the context of flagellar or pili glycosylation, however, recent studies have shown that the *O*-glycosylation systems in *Burkholderia cenocepasia* and *N. gonorrhoeae* can target multiple proteins [22,31]. Similarly, PilO from *Pseudomonas aeruginosa* was also found to target multiple proteins in *E. coli*, suggesting that it could glycosylate proteins other than pillins in *P. aeruginosa* [32]. Interestingly, the flagellin from *N. elongata* was found to be unglycosylated, in contrast to most flagellins studied to date [29].

### 2.6. O-Glycosylation by Sequential Action of Glycosyltransferases

In addition to the *en bloc O*-glycosylation systems, many bacteria encode enzymes that mediate mucin-type *O*-glycosylation, where the acceptor protein is modified intracellularly by the direct action of a GT, followed by extension of the glycan by the action of additional GTs. In its simplest form, the acceptor protein is modified, at the glycosylation site, by a single monosaccharide, with no further elongation of the glycan, as in the case of *C. jejuni* and *Campylobacter coli* flagellar glycosylation with single pseudaminic (Pse) or legionaminic acid (Leg) or their derivatives, respectively. In both cases, the genes encoding enzymes involved in the biosynthesis and subsequent transfer of the sugar onto the protein are located downstream of the *flaA* flagellin gene [33,34].

Examples of this *O*-glycosylation mechanism have also been described in a range of Gram-positive species. For example, strains of *Clostridium botulinum* glycosylate their flagella with a single hexuronic acid or Leg derivative per glycosylation site [35]. In contrast, *Clostridium difficile* uses two, more complex glycans to carry out protein glycosylation of its flagella. Type A glycans are composed of an *O*-GlcNAc modified with a Thr via a phosphodiester bond (Thr-P) and are synthesized by the sequential action of three enzymes (CD240, CD242 and CD243) [36,37]. Type B glycans consist of a β-*O*-GlcNAc, extended with two rhamnose (Rha) molecules, occasionally methylated, and capped with a unique sulfonated peptidyl fucosamine [38,39]. Three enzymes are involved in the addition of the monosaccharides onto the target protein, and an additional two enzymes synthesize the modified fucosamine [39] (see Figure 2B). *Bacillus antharacis* and the closely related *Bacillus cereus* glycosylate their spore protein with 3-*O*-Me-Rha-α-1,2-Rha-α-1,3-GalNAc, capped either with anthrose or cereose, respectively, which are sugars characteristic for each strain [40]. A distinct glycosylation system is found in *Listeria monocytogenes*, where the flagella is modified on several amino acids by a single β-*O*-linked GlcNAc [41]. The glycosidic linkage formed is similar to that found in the *C. difficile* glycosylation, but as the glycan is not further extended, it resembles the cytosolic *O*-GlcNAcylation mechanism that is involved in signaling pathways [42].

### 2.7. The Accessory Secretion System SecA_2_ Glycosylation Pathway

**Figure 3:**
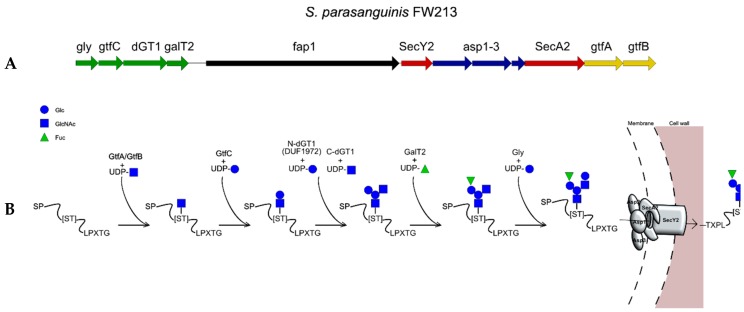
(**A**) The *secA_2_* cluster in *Streptococcus parasanguinis* FW213. (**B**) Fap1 glycosylation in *S. parasanguinis* FW213.

Several pathogenic Gram-positive bacteria possess an auxiliary secretion system (SecA_2_), in addition to the canonical SecA [43]. This system contains the necessary genes encoding proteins that facilitate the expression, glycosylation and subsequent secretion of serine rich repeat (SRR) containing proteins (SRRPs) [44]. The cluster contains variable number of GTs in different organisms; the best studied system is that of *Streptococcus parasanguinis* [45,46,47,48,49,50,51,52] which shows some unique features, not found in other glycosylation systems. In total, this cluster contains six GTs (see Figure 3). First, glycosylation of Fap1, the SRRP in *S. parasanguinis* FW213, is initiated by the combined action of two GTs, GtfA and GtfB. Please note that, in some studies, these have been referred to as Gtf1 and Gtf2, but for consistency we will refer to the SecA_2_ priming GTs as GtfA and GtfB throughout the review. These enzymes interact with the acceptor SRRP and with each other through a conserved domain DUF1975 and mediate the addition of the reducing GlcNAc. GtfA acts as a GT, whereas GtfB interacts with the acceptor protein as a chaperone [53,54]. GtfC, formerly annotated as sugar nucleotide synthase-like protein (NSS), extends the glycan by adding a Glc unit. dGT1 contains two distinct GT domains (DUF1792 in N-terminus, which is a recently described GT-D type GT-fold, and a GT-A type GT-fold in C-terminus [49] and creates a branching point by adding a Glc and a GlcNAc residue on Glc. GalT2 adds a Rha residue onto the second Glc and the glycosylation is completed by the addition of a Glc residue onto GlcNAc by Gly [52].

Interestingly, secretion of SRRPs through the SecA_2_ system does not depend on the glycosylation of SRR, but this modification blocks export through the canonical SecA system [46]. In addition, defective glycosylation of SRRP also leads to impaired binding of the respective bacteria onto model substrates and reduced virulence in mouse models [44,55,56].

## 3. Protein Glycosylation in Gut Commensal Bacteria

While protein glycosylation has been extensively studied in pathogens, underscoring the importance of glycoproteins in virulence and pathogenicity (for a recent review, see [57], the nature and function of protein glycosylation in gut commensal bacteria remains largely unexplored. However, due to the recognized importance of the role played by the gut microbiota in health and disease, the study of protein glycosylation in gut commensal bacteria is emerging as an expanding field of research.

Various glycoproteins have been identified in *Bacteroides fragilis*, a dominant member of the Bacteroidetes phylum considered to be a gut commensal bacterium. Most glycoproteins in cell lysates were found to be fucosylated, as shown using the fucose-specific *Aleuria aurantia* lectin (AAL). The bacteria could synthesize GDP-fucose from GDP-mannose, or acquire fucose (Fuc) from the growth media and activate it with GDP, after phosphorylation (both phosphorylation and subsequent activation are catalysed by Fkp) [58]. Affinity chromatography with AAL followed by mass spectrometry (MS) analysis identified glycoproteins of various functions, including peptidases, chaperones and proteins predicted to be involved in protein-protein interactions. All the identified proteins were predicted to be periplasmic or associated with the bacterial outer membrane [59]. It was also found that glycosylation took place in the periplasm, which suggested an *en bloc* glycosylation mechanism. Indeed, a gene cluster resembling a capsular polysaccharide (CPS) biosynthesis cluster was identified, which lacked a polymerase gene [59]. After its deletion, the affinity of the glycoproteins to AAL was lost, suggesting that this cluster plays a critical role in a general *O*-glycosylation system and that this system is independent of the CPS biosynthesis pathway [59]. Using an antibody specific for the *B. fragilis* glycan against protein extracts of various *Bacteroides* species, it was suggested that most of them, including *Bacteroides thetaiotaomicron* and *Bacteroides ovatus*, produce similar glycans. No glycosylation was observed in *Bacteroides vulgatus*, suggesting either a lack of glycosylation, or, more likely, a different glycan structure, as this bacterial species contains a homologous glycosylation system [59]. Interestingly, even though no consensus sequence has been identified for *O*-glycosylation, the *B. fragilis O*-OTase seems to be specific for the three-amino-acid long sequon Asp-(Ser/Thr)-(Ala/Ile/Leu/Met/Thr/Val). Mutation of the first Asp led to a loss of glycosylation in the proteins tested, and there was a clear requirement for an amino acid with at least one methyl group in its side chain in the position following the glycosylation site [60]. Based on this sequence, more than 1000 putative glycoproteins were identified in *B. fragilis*, and by introducing this sequence into a putative α-fucosidase from *B. fragilis*, which does not carry a glycosylation motif and naturally lack glycosylation, site-specific glycosylation was achieved in vivo [60].

Recently, SRRPs have also been reported to be glycosylated in *Streptococcus salivarius*, a pioneer colonizer and commensal bacterium of the human gastrointestinal (GI) tract [61]. In contrast to other Gram-positive bacteria which have a unique SRR glycoprotein-encoding gene (see above), *S. salivarius* expresses three large and glycosylated surface-exposed proteins—SrpA, SrpB and SrpC—that show characteristics of SRR glycoproteins and are secreted through the accessory SecA_2_ system. Two GTs, GtfE and GtfF, encoded outside of the *secA_2_* locus, unusually, perform the first step of the sequential glycosylation process, which is crucial for SRRP activity. SrpA, SrpB and SrpC are the main factors underlying the multifaceted adhesion of *S. salivarius* and, their glycosylation plays a major role in host colonization [61].

## 4. Protein Glycosylation in *Lactobacillus*

As *Lactobacillus* species have been extensively studied, owing to the importance of certain strains as probiotics, evidence for protein glycosylation has recently emerged.

### 4.1. Lactobacillus Glycoproteins

**Figure 4:**
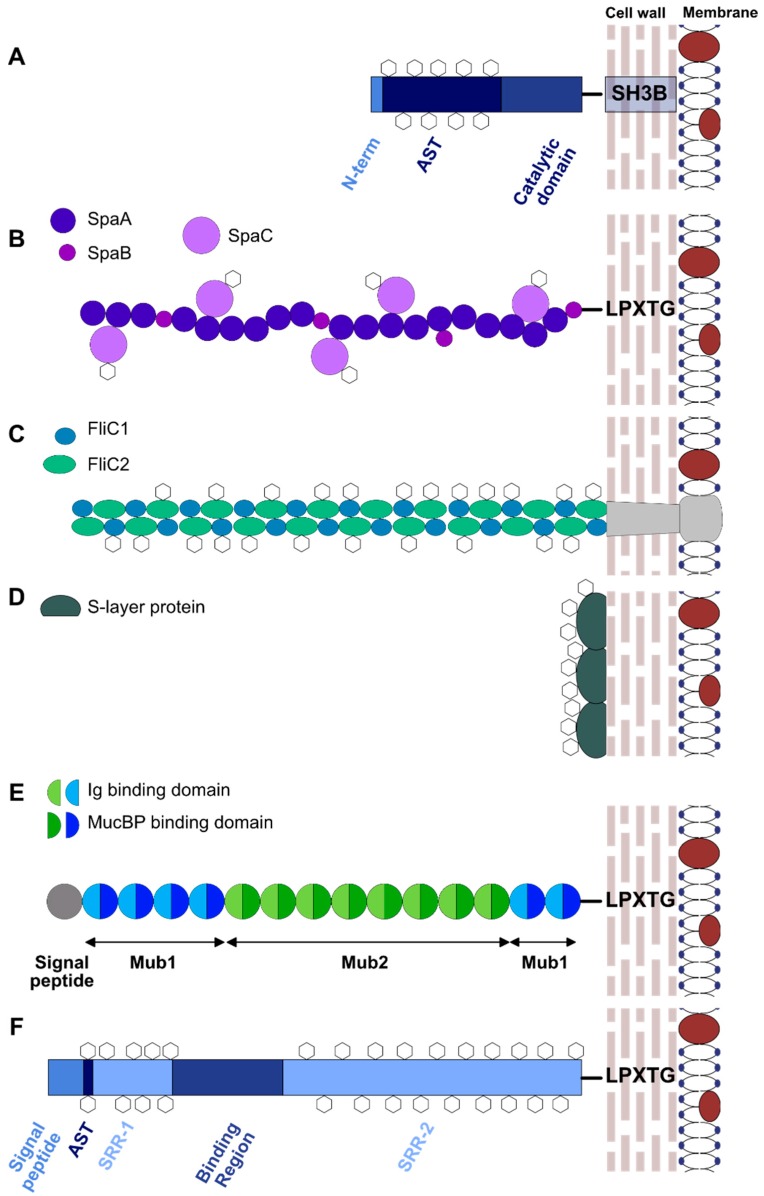
Schematic representation of glycosylated cell-surface proteins in *Lactobacillus* sp. (**A**) Muramidases, (**B**) SpaCBA pillus, (**C**) Flagellum, (**D**) S-layer proteins, (**E**) Mucus binding proteins. (**F**) Serine rich repeat proteins. The white hexagons represent the glycans found on the proteins.

Muramidases are the best characterized glycoproteins in *Lactobacillus* species. Acm2, the major autolysin of *Lactobacillus plantarum* strain WCFS1, is a modular protein. Its catalytic domain is surrounded by an *O*-glycosylated N-terminal region rich in Ala, Ser, and Thr (AST domain), which is of low complexity and unknown function, and a C-terminal region composed of five SH3b peptidoglycan binding domains (see Figure 4A. MS analysis showed that Acm2 is glycosylated by single *N*-acetylhexoseamine (HexNAc) residues at more than 20 glycosylation sites, all found within the AST domain [62]. This is in agreement with previous studies showing that *O*-glycosylation occurs in low complexity regions [22,31,57]. By deleting the secretion signal peptide of Acm2, Fredriksen et al. [62] showed that glycosylation occurs intracellularly and therefore precedes secretion [62]. It was also shown that glycosylation partially inhibited the enzymatic activity of Acm2 [63]. This was proposed to occur by interaction between the *N*-Acetylglucosamine (GlcNAc) moieties of the AST domain with either the active site, or the SH3b motifs, which are responsible for binding the GlcNAc-rich peptidoglycan layer [63]. Glycosylation also increased the resistance of the AST domain against trypsin [63].

Similar to Acm2, the major secreted protein 1 (Msp1) is a muramidase found to be glycosylated in *Lactobacillus rhamnosus* GG [64]. It has a predicted molecular weight (MW) of 48 kDa, but was found to migrate at 75 kDa on SDS-PAGE and interact with Concanavalin A (ConA), a lectin specific for mannose (Man) and Glc residues. Msp1 shows low complexity, as it consists of 23% Ala residues. Monosaccharide composition analysis of Msp1 confirmed the presence of Man, in agreement with ConA affinity to Msp1 [64]. As reported for Acm2, the glycosylation of Msp1 protected the protein against proteases. However, in contrast to Acm2, glycosylation of Msp1 did not affect the hydrolytic activity of the enzyme or its ability to activate the Akt signaling pathway in Caco-2 cells [64].

Interestingly, a muramidase from *Lactobacillus buchneri* CD034, belonging to family 25 of glycoside hydrolases (GH25) according to CAZy classification, and its homologue from *L. buchneri* NRRL B-30929 were also found to be glycosylated, with glycans consisting of eight glucose units, in a low complexity region [65].

Cell surface proteins (adhesins or lectins) play key roles in the adhesion of gut bacteria to the host tissue, especially the gut epithelium and mucus layer, by interacting with host proteins or glycoconjugates. These include (i) moonlighting proteins with various roles in the bacterial physiology (sometimes they also lack the signal peptide necessary for secretion), (ii) surface appendages such as pili and flagella, as well as (iii) specialized surface adhesins that bind to host tissue [66] (Figure 4A). Many of these proteins have been shown to be glycosylated (see Table 1).

Pili and flagella are large polymeric proteins that form long surface structures which are involved in bacterial adhesion. Although rare in Gram-positive bacteria, pili have been identified in *L. rhamnosus* GG, where they confer binding to mucus [71] and are predicted to exist in other *Lactobacillus* species, based on genomics analyses [72]. In *L. rhamnosus* GG, these are composed of the three-protein complex SpaCBA, which is assembled by a pilin-specific sortase [73] (see Figure 4B). The SpaCBA proteins have been involved in adhesion to intestinal epithelial cells (IEC) and in the attenuation of proinflammatory responses from these cells [74]. Atomic force spectroscopy (AFM) of the pili using functionalized tips with lectins specific for Man and Fuc suggested the presence of these two monosaccharides, in contrast to the glycosylation analysis of *L. rhamnosus* GG Msp1, which only detected the presence of Man residues and no Fuc. Furthermore, the glycosylated pili were shown to interact with dendritic cells (DCs) via the DC-SIGN (Dendritic Cell-Specific Intercellular adhesion molecule-3-Grabbing Non-integrin) lectin, an important receptor of the immune system that recognizes primarily high-mannose structures, and induce the expression of the anti-inflammatory cytokine IL-10, as well as IL-6 and IL-12p35 [67]. Flagellar proteins have been extensively studied in pathogens such as enteropathogenic and enterohemorrhagic *E. coli* and *Campylobacter* sp. where they have been shown to be important components of adhesion to host tissue [75,76]. Only a few *Lactobacillus* species have the genetic potential to produce flagella which can induce pro-inflammatory responses by the host [77]. These were recently characterized in a motile strain of *Lactobacillus agilis* and shown to be glycosylated by periodic acid/Schiff (PAS) staining [68] (see Figure 4C), but the nature of glycosylation was not investigated further.

Surface Layer Proteins (Slps) comprise the majority of the bacterial surface protein load and play key roles in aggregation and binding to mucus or the extracellular matrix (ECM) [69,72]. Slps are expressed by many bacterial species and form a two-dimensional (2-D) layer that surrounds the bacterial cells [78]. In Gram-positive bacteria, Slps are found attached onto components of the peptidoglycan (PG) layer, such as (lipo)teichoic acids or neutral polysaccharides [79] (see Figure 4D). In *Lactobacillus* species, these proteins usually consist of a *C*-terminal carbohydrate-binding domain, used for attachment of the protein onto the cell wall, and a self-assembly N-terminal domain that forms the 2D layer [80]. Although glycosylated Slps from *Lactobacillus helveticus* ATCC12046 [81] and *L. plantarum* 41021/252 [82] had been shown to be detected by PAS staining, *Lactobacillus* Slps were generally considered to be non-glycosylated [69]. However, recent studies of *Lactobacillus kefir* [69], *Lactobacillus acidophilus* [83] and *Lactobacillus buchneri* [65] strains revealed more glycosylated S-layer proteins. SlpA in *L. acidophilus* NCFM was found to be glycosylated with glycans containing Man and Fuc, as shown by AFM experiments with specific lectins [83]. Similar to SpaCBA pili from *L. rhamnosus* GG, SlpA from *L. acidophilus* NCFM induced the production of IL-10 from DC, by interacting with DC-SIGN [83]. MS analysis of SlpA from *L. buchneri* CD034 showed that the protein is glycosylated on serine residues in the sequon Ser-Ser-Ala-Ser-Ser-Ala-Ser-Ser-Ala, consistent with previous reports for *O*-glycosylation in low complexity and AST-rich regions [22,31,62]. The glycans found on each glycosylation site consisted of 7 residues of α1–6 linked Glc on average. It was also suggested that glycosylation occurs extracellularly, as no glycosylated SlpA was found in the cytosolic fraction [65]. Since this glycosylation profile is similar to the one reported for the GH25 muramidases from these strains (see above), it is possible that SlpA and the muramidases are modified by the same glycosylation system [65]. Screening of various *L. kefir* strains showed that Slp glycosylation is conserved within this species [69], but the nature of the glycan or the glycosylation mechanism remain unexplored.

Mucus Binding proteins (MUBs) containing Mub repeats have been identified primarily in lactic acid bacteria [72] and are more common in those colonizing the GI tract [84]. MUB from *L. reuteri* ATCC 53608 is one of the best characterized examples of mucus adhesins in commensal bacteria [85]. MUB presents a C-terminal LPxTG anchoring motif, and an N-terminal secretion signal peptide. It is a high molecular weight protein that consists of six type 1 Mub (Mub1) repeats and eight type 2 Mub repeats (Mub2), based on sequence homology (see Figure 4E). Each repeat is divided into two domains, a mucin binding (MucBP) domain and an immunoglobulin binding (Ig-binding protein) domain [84,86]. The Mub repeats mediate binding to mucin glycans, through interactions with terminal sialic acid [86,87], and immunoglobulins [84]. MUB has the shape of a long, fiber-like structure, of around 180 nm in length [88], and forms appendices similar to pili found in pathogenic and, more rarely, other commensal bacterial species. However, it has been shown by force spectroscopy, that in contrast to many pathogenic adhesins which show binding capacity at the N-terminal tip, the particular structural organization of MUB maximizes interactions with the mucin glycan receptors through its long and linear multi-repeat structure [87]. This multivalent binding is in agreement with the location/confinement of commensal bacteria within the outer mucus layer in the large bowel. In addition, MUB from *L. reuteri* ATCC53608 was recently shown to interact with DC-SIGN, leading to increased levels of pro-inflammatory cytokines and CD83 [89]. This lectin recognition in addition to its aberrant electrophoretic mobility [85], suggests that MUB may be glycosylated. Proteins containing one or more copies of MucBPs have been identified across most *Lactobacillus* species, as well as proteins containing Mub repeats [66,88]. MS analysis of surface proteins in *L. plantarum* WCFS1 revealed proteins carrying *O*-GlcNAc residues as shown for Acm2, including a mucus binding protein, similar to MUB from *L. reuteri* ATCC 53608, and therefore probably involved in adhesion of the bacteria to the host surface [70].

### 4.2. Protein Glycosylation Pathways in Lactobacillus

The best studied example of protein glycosylation pathway in *Lactobacillus* is from *L. plantarum* WCFS1, where a general glycosylation system has been described [90]. In addition to the well-characterized glycosylated Acm2 muramidase (see above), MS analysis of surface proteins in *L. plantarum* WCFS1 revealed numerous proteins carrying *O*-GlcNAc residues, including DnaK, a chaperone involved in protein folding, PdhC, which is involved in the anaerobic metabolism of Lactobacilli, as well as a mucus binding protein [70] (Table 1). The glycosylation mechanism in *L. plantarum* WCFS1 is similar to the initiating glycosylation pathway of SRRPs in pathogens, where two GTs, named as Gtf1 and Gtf2 in *L. plantarum* (originally named GtfA and GtfB, but referred to as Gtf1 and Gtf2 here, so as to avoid confusion with the SecA_2_ specific GTs), are involved in the addition of a single HexNAc molecule on the glycosylation site of the acceptor proteins [90]; deletion of either of these genes led to a loss of recognition of the glycoproteins by the wheat germ agglutinin (WGA) lectin. This suggests that both enzymes are required for protein glycosylation and that the added sugar is most likely GlcNAc. These two enzymes contain a DUF1975 in N-terminus which probably mediates the interaction between the two GTs and the target proteins and a GT domain in C-terminus, suggesting a similar mode of action to the SecA_2_ specific GtfA and GtfB (see above).

This analysis also identified a HexNAc-Hex moiety on γ-d-glutamate-meso-diaminopimelate muropeptidase, which suggests that either the GlcNAc residues can be further extended by the action of other GTs, or that there is an additional glycosylation system in *L. plantarum* [70]. In addition to WGA, *Dolichos biflorus* agglutinin, a lectin specific for α-GalNAc, and *Lens culinaris* lectin, which is specific for α-mannose, were also shown to interact with *L. plantarum* proteins [90]. This would also suggest the presence of additional glycosylation system(s). However, deletion of four other putative GTs (including one with a DUF1975), similar to GtfA from *S. parasanguinis* in *L. plantarum* WCFS1 did not lead to any changes in the recognition of the proteins by these lectins [90].

Analysis of protein glycosylation in *L. rhamnosus* GG revealed that the heterotrimeric pili and Msp1, are glycosylated. However, information on the protein glycosylation pathway of this strain is limited. While *L. rhamnosus* GG contains a pair of putative glycosyltransferases containing a DUF1975, these have not been experimentally assessed for their involvement in glycosylation of either Msp1 or the SpaCBA pilin.

**Figure 5:**
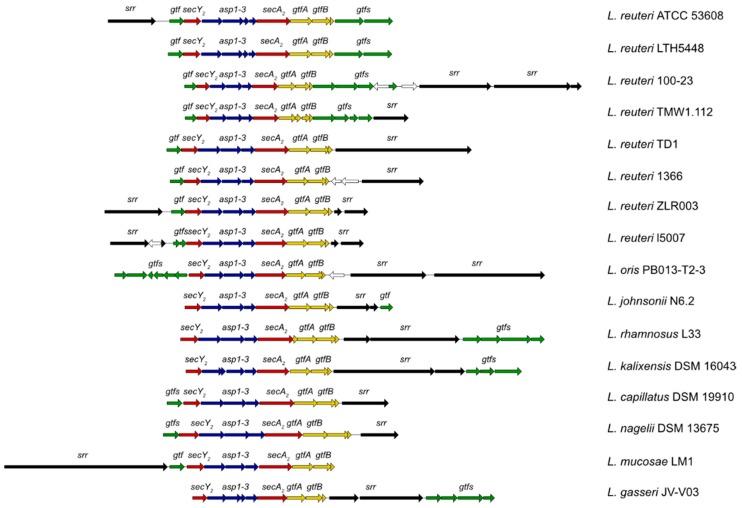
Organization of the *secA_2_* clusters identified in *Lactobacillus* genomes. The genes encoding the translocases SecA_2_ and SecY_2_ are shown in red, the accessory secretion proteins asp1–3 in blue and the priming GTs, GtfA and GtfB, in yellow. Genes encoding additional GTs are shown in green and the genes encoding serine-rich repeat proteins are illustrated in black. White arrows represent genes that are not part of the SecA_2_ machinery.

To date, *secA*_2_ clusters have been identified in the genomes of various *Lactobacillus* species [91]. This accessory secretion system is dedicated to the glycosylation and secretion of SRRPs. The secretion system typically consists of two translocases, SecA2 and SecY2, three accessory Sec system proteins (asp1–3), and a variable number of GTs, ranging between three to seven (see Figure 5).

The various SRRPs are divided into distinct subdomains: a cleavable and unusually long signal peptide which, in some cases, is followed by an alanine-serine-threonine rich (AST) motif, a short serine rich repeat region, a binding region (also known as “basic region” due to its unusual composition of basic amino acids), a second and much larger serine rich repeat region, and a cell wall anchoring motif [92]. In *L. reuteri* strains, the cluster has only been found in isolates of murine or porcine origins, and it appears to be absent from isolates of human origin [91,93]. The cluster in the murine isolate *L. reuteri* 100-23 is crucial for adhesion of the bacteria to the forestomach epithelium of the murine GI tract, as shown by colonization experiments in mice with *L. reuteri* 100-23 and mutants lacking putative adhesins [94]. Mutants lacking the *secA_2_* gene showed defective adhesion, whereas mutants lacking *srr* showed the most reduced colonization, compared to other putative adhesins tested [94]. However, the nature of SRRP glycosylation in *Lactobacillus* strains has not yet been reported at the protein level.

## 5. Conclusions

Host-microbe interactions in the gut influence the outcome of pathogenic infection or commensal colonization and are thus key to gut homeostasis. Although the importance of protein glycosylation is now widely acknowledged in pathogens, more effort is needed to gain a better understanding of glycosylation in our resident gut bacteria. Glycoproteins in particular are an under-studied, potentially crucial factor underpinning bacteria-host interactions including adhesion, biofilm formation and immune response. Glycosylation increases protein diversity and structure, and, as such, significantly impacts on the function of the resulting glycoprotein including the ability for some bacteria to co- or auto-aggregate. Recent advances in new structural and analytic tools, glycomics, will facilitate further investigations into novel bacterial glycan structures and characterization of glycosylation pathways in major gut commensal bacteria. Such knowledge is required to understand the role of protein glycosylation in determining the fate of bacteria–host interactions and to fully exploit the increasing range of bacterial molecules involved in the development of novel therapeutic approaches (drugs and biomarkers) targeting the microbiome.

## Figures and Tables

**Table 1:** Summary of the main glycoproteins identified and characterized in *Lactobacillus* species.

Protein	Organism	Glycan	Method *	Reference
Msp1	*L. rhamnosus* GG	Man-containing	Pro-Q Emerald stain, Lectin affinity (WB, AFM) MS	[64]
SpaCBA	*L. rhamnosus* GG	Man and Fuc-containing	Lectin affinity (AFM, WB, ELLA)	[67]
FliC_1_/FliC_2_	*L. agilis*	uncharacterized	PAS-stain	[68]
SlpB/N	*L. buchneri* CD034, *L. buchneri* NRRL B-30929	Glc_1_–Glc_7_	MS	[65]
LbGH25B/N Putative glycosyl-hydrolase	*L. buchneri* CD034, *L. buchneri* NRRL B-30929	Glc_8_	MS	[65]
Slp	*L. kefir*	uncharacterized	PAS stain	[69]
Acm2	*L. plantarum* WCFS1	GlcNAc	MS, lectin affinity (WB)	[62]
DnaK	*L. plantarum* WCFS1	GlcNAc_1_, GlcNAc_1_Hex_1_	MS	[70]
Lp_2162 (muropeptidase)	*L. plantarum* WCFS1	GlcNAc_1_	MS	[70]
Lp_2260	*L. plantarum* WCFS1	GlcNAc_1_	MS	[70]
Lp_1643 (mucus binding protein)	*L. plantarum* WCFS1	GlcNAc_1_	MS	[70]
PdhC	*L. plantarum* WCFS1	GlcNAc_1_	MS	[70]
FtsY	*L. plantarum* WCFS1	GlcNAc_1_	MS	[70]
Lp_2793	*L. plantarum* WCFS1	GlcNAc_1_	MS	[70]
FtsK1	*L. plantarum* WCFS1	GlcNAc_1_	MS	[70]
Lp_3421 (muropeptidase)	*L. plantarum* WCFS1	GlcNAc_1_, GlcNAc_1_Hex_1_	MS	[70]
FtsZ	*L. plantarum* WCFS1	GlcNAc_1_	MS	[70]

* Abbreviations: WB, western blot, AFM, atomic force microscopy, MS, mass spectrometry, PAS, periodic acid/Schiff, ELLA, Enzyme-linked lectin assay.
